# Effectiveness of artemisinin-based combination therapy used in the context of home management of malaria: A report from three study sites in sub-Saharan Africa

**DOI:** 10.1186/1475-2875-7-190

**Published:** 2008-09-27

**Authors:** Ikeoluwapo O Ajayi, Edmund N Browne, Fred Bateganya, Denis Yar, Christian Happi, Catherine O Falade, Grace O Gbotosho, Bidemi Yusuf, Samuel Boateng, Kefas Mugittu, Simon Cousens, Miriam Nanyunja, Franco Pagnoni

**Affiliations:** 1 Malaria Research Laboratories, Institute of Medical Research and Training, College of Medicine, University of Ibadan, Nigeria; 2 Department of Community Health, School of Medical Sciences, KNUST, Kumasi, Ghana; 3 Department of Sociology, Makerere University, Kampala, Uganda; 4 Ifakara Health Institute (IHI, P.O. Box 53, Ifakara and P.O. Box 78373, Dar es Salaam, Tanzania); 5 Infectious Diseases Epidemiology Unit, London School of Hygiene and Tropical Medicine, London, UK; 6 WHO Country Office, Uganda; 7 Evidence for Antimalarial Policy and Access Unit, UNICEF/UNDP/World Bank/WHO Special Programme for Research and Training in Tropical Diseases (TDR), Geneva, Switzerland

## Abstract

**Background:**

The use of artemisinin-based combination therapy (ACT) at the community level has been advocated as a means to increase access to effective antimalarial medicines by high risk groups living in underserved areas, mainly in sub-Saharan Africa. This strategy has been shown to be feasible and acceptable to the community. However, the parasitological effectiveness of ACT when dispensed by community medicine distributors (CMDs) within the context of home management of malaria (HMM) and used unsupervised by caregivers at home has not been evaluated.

**Methods:**

In a sub-set of villages participating in a large-scale study on feasibility and acceptability of ACT use in areas of high malaria transmission in Ghana, Nigeria and Uganda, thick blood smears and blood spotted filter paper were prepared from finger prick blood samples collected from febrile children between six and 59 months of age reporting to trained CMDs for microscopy and PCR analysis. Presumptive antimalarial treatment with ACT (artesunate-amodiaquine in Ghana, artemether-lumefantrine in Nigeria and Uganda) was then initiated. Repeat finger prick blood samples were obtained 28 days later for children who were parasitaemic at baseline. For children who were parasitaemic at follow-up, PCR analyses were undertaken to distinguish recrudescence from re-infection. The extent to which ACTs had been correctly administered was assessed through separate household interviews with caregivers having had a child with fever in the previous two weeks.

**Results:**

Over a period of 12 months, a total of 1,740 children presenting with fever were enrolled across the study sites. Patent parasitaemia at baseline was present in 1,189 children (68.3%) and varied from 60.1% in Uganda to 71.1% in Ghana. A total of 606 children (51% of infected children) reported for a repeat test 28 days after treatment. The crude parasitological failure rate varied from 3.7% in Uganda (C.I. 1.2%–6.2%) to 41.8% in Nigeria (C.I. 35%–49%). The PCR adjusted parasitological cure rate was greater than 90% in all sites, varying from 90.9% in Nigeria (C.I. 86%–95%) to 97.2% in Uganda (C.I. 95%–99%). Reported adherence to correct treatment in terms of dose and duration varied from 81% in Uganda (C.I. 67%–95%) to 97% in Ghana (C.I. 95%–99%) with an average of 94% (C.I. 91%–97%).

**Conclusion:**

While follow-up rates were low, this study provides encouraging data on parasitological outcomes of children treated with ACT in the context of HMM and adds to the evidence base for HMM as a public health strategy as well as for scaling-up implementation of HMM with ACTs.

## Background

Artemisinin-based combination therapy (ACT) is currently the universally recommended first-line therapy for uncomplicated malaria [1]. Numerous trials have shown ACT to be highly efficacious when used under controlled conditions [2-4], and some studies have confirmed their efficacy when used under partially supervised conditions [5,6].

The use of ACT at the community level has been advocated as a means to increase access to effective antimalarial medicines by high risk groups living in underserved areas, mainly in sub-Saharan Africa [1,7,8]. This strategy, known as Home Management of Malaria (HMM), has been shown to be feasible and acceptable to the community [9-14]. However, while wide deployment of ACT at the community level through HMM has the potential to greatly reduce malaria related mortality and severe morbidity, such implementation has until now been delayed by substantial economic and public health issues. These include concerns about the effective use of ACT at the community level [15], as the parasitological cure rate that is achieved with ACT in the context of HMM, when dispensed by community medicine distributors (CMDs) and used unsupervised by caregivers at home, has not been assessed.

In addition, poor adherence to the treatment schedule by both caregivers and CMDs, or a failure to provide adequate storage conditions to ensure drug stability in the community could reduce the efficacy of ACT and facilitate the emergence of drug resistance. There is thus a need for research to provide the evidence base to accompany deployment of ACT beyond the health facility level [16,17] including documenting the actual cure rate that can be obtained by ACT deployed within the context of HMM programmes.

To provide additional evidence to support the use of ACT at the community level, the UNICEF-UNDP-WORLD BANK-WHO Special Programme for Research and Training in Tropical Diseases (TDR) funded studies in Ghana, Nigeria and Uganda, representing different health systems and epidemiological settings. Report on the feasibility and acceptability of incorporating ACT in HMM has been published [18]. In this paper the parasitological cure rates achieved by ACT provided at the community level within the context of HMM programmes in the same study sites are reported.

## Methods

A multi-centre study of the use of ACT within the context of HMM in three African countries was carried out. Details of the study sites and the implementation of HMM in those sites have been previously reported [18].

### Study sites

Three sites participated in the evaluation of parasitological cure rates: Ejisu-Juaben district in Ghana, Badeku and Ojoku/Ajia districts in Nigeria, and Bugiri district in Uganda. All three sites are rural areas in which malaria is hyper-endemic, with perennial transmission accompanied by seasonal peaks. The entomological inoculation rate (EIR) is estimated to be of the order of 500 infective bites per person per year in Ejisu-Juaben district [19] and Badeku and Ojoku/Ajia districts [20]. The EIR is not known for Bugiri district in Uganda, but is reported to be 500 infective bites per person per year or more in the neighbouring district of Tororo [21], which has similar ecology. In each site, a sub-sample of all villages participating in the study was selected for the study of ACT effectiveness, based on accessibility during the rainy season and logistics: 9/36 villages in Ghana, 7/40 in Nigeria and 3/37 in Uganda.

### Study design

The study involved follow-up of children between six to 59 months of age, whose caregivers reported to CMDs with a complaint of fever. The CMDs in the study areas had been trained on the signs and symptoms of both severe and uncomplicated malaria, to educate caregivers on the use of ACTs, on the preparation of thin and thick blood films and blood spots on filter paper as well as on proper storage of the specimens (filter papers and stained blood smear slides).

### Study implementation

At the time of the first visit of the febrile child and after informed consent had been obtained from the caregiver , CMDs prepared blood slides for microscopy and collected blood on filter paper [3 mm Whatman] for PCR analysis. In line with the currently recommended HMM strategy [8]), antimalarial treatment with ACT was then given based on clinical grounds. Children with signs of severe disease were excluded from the study and immediately referred to the nearest health centre. In Ghana and Nigeria antimalarial drugs were given to caregivers accompanied by instructions, following which caregivers treated their children at home unsupervised. In Uganda, the first dose was administered to the child in the presence of the CMD, while subsequent doses were administered unsupervised, although the CMDs were expected to make home visits to remind the mothers to give the treatment on subsequent days. All caregivers were instructed to report to the CMDs or the nearest health facility if the child had not improved after 48 hours or if the child deteriorated at any time.

Blood slides were read daily in local research laboratories and results returned to the CMDs the next day. Thereafter, CMDs provided the result to the mothers/caregivers. Mothers/caregivers of children with microscopically confirmed malaria were instructed to return for follow-up on day 28. On day 28 the caregivers were asked about symptoms and repeat peripheral blood films and blood spots on filter paper were obtained from their children. Caregivers of children who were free of patent parasitaemia were advised to take their children to the health centre or any health facility of their choice.

### Study drugs

In accordance with the national drug policies of the participating countries, a fixed combination of 20 mg of artemether and 120 mg of lumefantrine (AL) was used in Uganda and Nigeria, and a loose combination of artesunate and amodiaquine (AS/AQ) was used in Ghana. The recommended dose for AL in children between six and 35 months of age was one tablet twice daily for three days, and two tablets twice daily for three days in children between 36 and 59 months of age. Caregivers were advised to administer the drug after meals, preferably fatty food. For the AS/AQ combination, blister packs containing co-packed artesunate and amodiaquine tablets of 50 and 153 mgs respectively were used. For children aged 12 to 59 months the recommended dose of AS/AQ was one tablet of each drug once per day. However, for children aged 6 to 11 months, tablets had to be broken in half and repackaged to comply with the recommended AS/AQ dose in that age group. In all countries, antimalarial medicines for the study were provided by the Ministry of Health free of charge.

### Training

#### Recruitment and training of field supervisors

A two-day training workshop was organized for the field supervisors to acquaint them with the intervention activities and the data collection tools. They were trained on how to make slides, blood spots on filter paper for PCR analysis and on proper storage of the specimens (filter papers and stained blood smear slides) in order to adequately supervise the CMDs. The mechanisms of drug distribution and supervision were also discussed.

#### Recruitment and training of the community based medicine distributors

A total of 47 CMDs were recruited (17 in Ghana, 24 in Nigeria and 6 in Uganda), following selection by the respective communities. A three-day workshop (two days of demonstration followed by a one day field practical) was held for the CMDs in each study site. The training covered the clinical features of malaria, diagnosis, preparation of blood smears on slides, spotting blood on filter paper and treatment including dosage regimen. The CMDs were also instructed on the administration of consent forms, filling of data collection forms and criteria for referral. Refresher training on slide and filter paper preparation was performed during supervision by the field staff in the first month after initial training.

#### Caregivers' compliance and CMDs' correctness of prescription

Adherence to prescribed treatment schedules was assessed through cross-sectional surveys involving household interviews with caregivers of children with fever in the previous two weeks. All households in the study communities were visited and caregivers who met the inclusion criteria [has a child aged 6–59 months with fever in the two weeks preceding survey and have been resident in the community for at least six months] were interviewed. In cases were more than one caregiver in a household met the inclusion criteria, balloting was carried out to select the one to interview. During the interview, caregivers were questioned about the timing of treatment, the number of doses administered and the number of days over which drug was given. Used packs of drugs were inspected where available. The correctness of prescription by the CMDs was assessed by inspection of CMDs' registers during supervisory visits.

### Laboratory procedures

#### Microscopy

Thick blood films and Whatman filter papers with blood spots prepared by CMDs from finger prick blood samples were collected from the CMDs within 24 hours. Blood films were stained with 10% Giemsa stain for 30 minutes and screened microscopically under X100 oil immersion lens using a light microscope. Intensity of infection was estimated by counting the number of asexual parasites in relation to 200 white blood cells. Parasite density was calculated assuming a mean leukocyte count of 8,000/μl blood. Ten percent of the slides were selected at random and re-read by an independent microscopist at each centre. Disagreements in readings were found in less than 5% of cases in all sites.

#### DNA extraction and PCR amplification

Molecular genotyping for all sites was performed in Ibadan, Nigeria. Parasite genomic DNA was extracted from filter paper blood spots using the chelex extraction method [22]. Genotyping of parasite populations was performed for all samples collected from patients with microscopically confirmed *Plasmodium falciparum *infections at enrollment and who were parasitaemic at day 28 follow-up. Three highly polymorphic loci (block 1 of merozoite surface protein 1 (*msp1*), block 3 of merozoite surface protein 2 (*msp2*) and region II of glutamate rich protein (*glurp*)) of *P. falciparum *were amplified by nested PCR using oligonucleotide primers, reaction conditions and procedures described previously [22]. Ten microliters of each of the nested PCR products was resolved by electrophoresis on a 2% agarose gel stained with ethidium bromide and sized against a 100-base pair molecular weight marker (New England Biolabs, Beverly, MA).

Each *P. falciparum *infection was characterized on the basis of fragment sizes of PCR products for each locus. If an isolate had just one allele at each of the three loci, the clone number was taken to be one. Infections were defined as polyclonal if more than one allele from one or more genes was apparent, as previously described [23] and currently recommended by the WHO/MMV informal consultation on methods and techniques for genotyping to identify parasite population [24]. Briefly, recrudescent infections were defined as the occurrence of the same or a subset of alleles at all three loci (*msp-1*, *msp-2 *and *glurp*) in pre- and post-treatment samples. A lack of allelic identity at any of the three loci in pre- and post-treatment samples was considered to indicate a newly acquired infection/re-infection. An "unresolved" result was recorded when the parasite DNA could not be amplified. These samples were excluded from the analysis.

### Data analysis

Data were entered onto microcomputer and analysed using SPSS version 12.0 (SPSS Inc., Chicago, IL, USA) and STATA 9.0 (Stata Corp., Texas, USA 2005)

### Ethical clearance

The study was approved by the Ethics Review Committees in all participating countries and at the World Health Organization (WHO).

## Results

### Parasite prevalence and cure rate

Over a period of 12 months, a total of 1740 children presenting with fever were enrolled across the study sites. Of these, 1,189 (68.3%) had patent parasitaemia prior to treatment, of whom 606 (51%) reported for follow-up 28 days after treatment. The prevalence of parasitaemia prior to treatment was lower in Uganda than in Nigeria and Ghana (61% vs. 71% and 71% respectively, p < 0.001). The proportion of children followed up at Day 28 was lowest in Ghana (34%) compared with 60% in Nigeria and 78% in Uganda. The crude parasitological failure rate was 41.8% in Nigeria (C.I. 35%–49%), 27.8% in Ghana (C.I. 22%–34%) and 3.7% in Uganda (C.I. 1.2%–6.2%). The PCR adjusted cure rate was over 90% in all sites, ranging from 91% in Nigeria and Ghana (C.I. 86%–95% and 87%–95% respectively) to 97% in Uganda (C.I. 95%–99%) (Table 1).

**Table 1:** Treatment outcome of AL and AS/AQ at D28.

Study site	Drug used	Enrolled	Parasitaemic patients on D0 N (%)	Patients seen at D28 N (%)	Crude parasitological failure (%)	Geometric mean parasite density D28 (p/μl)	PCR adjusted failure rate*	PCR adjusted cure rate
Badeku & Ojoku/Ajia Nigeria	AL	432	306 (70.8%) C.I. 67%–75%	184 (60.1%)	77/184 (41.8%) C.I. 35%–49%	2835	14/154 (9.1%)	90.9% C.I. 86%–95%
Ejisu-Juaben, Ghana	AS/AQ	848	603 (71.1%) C.I. 68%–74%	205 (34%)	57/205 (27.8%) C.I. 22%–34%	15080	17/197 (8.6%)	91.4% C.I. 87%–95%
Bugiri, Uganda	AL	460	280 (60.9%) C.I. 56%–65%	217 (77.5%)	8/217 (3.7%) C.I. 1.2%–6.2%	N/A	6/216 (2.8%)	97.2% C.I. 95%–99%

*= Samples that could not be classified by PCR were excluded from the analysis

AL = artemether-lumefantrine

AS/AQ = artesunate-amodiaquine

### Adherence

Adherence by caregivers to the correct treatment was assessed in 244 febrile children across the three study sites. Adherence was measured in terms of caregiver's report of the number of doses administered to the child, the number of days over which the treatment was given and the promptness of treatment after onset of symptoms. Overall, 94% (C.I. 91%–97%) of children were treated correctly in terms of drug dose and duration of administration. The level of adherence was lower in Uganda (81%, C.I. 67%–95%) compared to Nigeria (93%, C.I. 87%–99%) and Ghana (97%, C.I. 95%–99%) (Table 2).

**Table 2:** Adherence of caregivers to treatment schedule in terms of dose and duration.

	Ejisu – Juaben District, Ghana	Badeku and Ojoku/Ajia districts, Nigeria	Bugiri District, Uganda	Totals
Total number of febrile children identified	211	132	44	387
Number of febrile children treated with ACTs from a CMD	153	60	31	244
Number (%) of children correctly treated (dose and duration)	149 (97%) **C.I. 95%–99%**	56 (93%) **C.I. 87% – 99%**	25 (81%) **C.I. 67% – 95%**	230 (94%) **C.I. 91% – 97%**

(source: HH survey in effectiveness sites)

## Discussion

The study provides two important new pieces of information. Firstly, it quantifies the prevalence of malaria infection among children seeking treatment from CMDs because of fever of suspected malaria origin in three areas of high malaria transmission. In all study sites (all rural, with EIRs of the order of 500) malaria prevalence exceeded 50%, ranging from 61% to 71%. This finding has important implications for policy at local level, as the usefulness and cost-effectiveness of rapid diagnostic tests (RDT) for malaria may vary with the intensity of transmission [25] and with the parasitaemia prevalence level [26]. The proportion of children with parasitaemia in this study is higher than that of 42% reported in Tanzania in a population of febrile children seeking treatment from drug stores or health facilities [27]. However, the Tanzania study did not report the EIR in that area.

Secondly, it provides the first data on the cure rate that can be obtained with the use of ACT prescribed by CMDs and administered unsupervised by caregivers at home, a strategy that is advocated to improve access to effective treatment by underserved communities living in sub-Saharan Africa. The PCR adjusted cure rate varied among the sites from 91% in Nigeria to 97% in Uganda, rates which are all above the threshold of 90% below which WHO recommends changing antimalarial drug policy [28]. However, the failure rates recorded in the Nigeria and Ghana sites (9.1% and 8.6% respectively) are higher than those reported in controlled AL and AS/AQ efficacy studies (< 5%) [4,29], while that recorded for Uganda (2.8%) is consistent with reports in literature. The study also shows that at Day 28, the majority of infections that can be detected by microscopy after treatment with AL or AS/AQ are due to re-infections with new strains of *P. falciparum*, a finding that had already been reported in literature [23].

Incomplete adherence by caregivers to the correct dosage and treatment regimen when unsupervised, has been suggested as a possible cause of decreased effectiveness of ACT [30,31] and a concern with regard to HMM with ACT [15]. While some studies failed to find a difference in cure rate between ACT dispensed supervised and unsupervised [5,6], others have reported a significant difference in therapeutic response after supervised and unsupervised treatment that can only be explained by insufficient patient adherence [30].

This seems an unlikely explanation in this study. Reported adherence by caregivers was consistently high (over 80%) across sites. However, adherence was assessed based on caregivers' recall only and the possibility that some caregivers did not report adherence accurately should be borne in mind. Day 7 plasma lumefantrine level has been reported to be a useful predictor of the bioavailability and compliance with the artemether lumefantrine co-formulation [31,32]. Thus, measurement of drug plasma levels may offer a more reliable method of measuring adherence than recall but is more invasive and more costly.

Higher crude parasitological failure rates were observed in Nigeria and Ghana compared to Uganda, despite the former areas reporting higher proportions of correctly treated children than the latter. Although the rate of recrudescence was drastically reduced in both Nigeria and Ghana after genotyping, the overall pattern remained unchanged (Table 1). This pattern might be partly explained by higher transmission intensity in Nigeria and Ghana compared to Uganda, as suggested by higher baseline parasite prevalence in Table 1. It should also be noted that the frequency and distribution of *msp1*, *msp2 *and *glurp *alleles used to correct the failure rate have not been thoroughly investigated in the study areas. As in many other studies [33-35], these alleles were used under the assumption that they have similarly high levels of diversity across the study sites. If there is little genetic diversity, but high transmission intensity, new infections of the same genotype will be common and erroneously classed as recrudescence. Therefore, it is recommended that genotyping results in efficacy studies and clinical trials should be interpreted with some caution [23].

In interpreting these results, it is important to consider some limitations of the study methods. The protocol used to determine the parasitological efficacy of ACT is simple, with collection of blood samples done only at baseline and after 28 days of treatment. This has limited the capacity to detect the timing at which treatment failures occurred and to analyse the relative contribution of recrudescences and re-infections over time. Also, the follow-up procedure of children with malaria positive microscopy at baseline was mainly based on spontaneous reporting, which may have introduced a selection bias in the group of children that were tested at Day 28, with important variations in the proportion of children reporting for follow up across study sites. Finally, adherence was measured by questionnaires administered to caregivers, providing potential for recall bias, and no determination of lumefantrine blood levels was done. Most of these limitations derive from the fact that the study was carried out as part of a larger study to determine feasibility and acceptability of ACT use within HMM, including caregiver adherence and treatment coverage. In this context there was a need to minimize interference with spontaneous behaviours and real life conditions of use of antimalarials. A strict follow up protocol would have influenced the behaviour of the caregivers and thus limited the generalizability of the findings of the main study.

The community effectiveness of antimalarial treatment is known to be influenced by a variety of factors beyond parasitological efficacy. These include access to effective treatment, quality of prescription and caregiver adherence [18,36-38]. However, measuring the parasitological cure rate that can be obtained using ACT in the context of HMM is of fundamental importance, given the current call for widespread use of ACT at community level to move towards malaria elimination [7]. While further studies, carried out under more controlled conditions, are necessary to establish the cure rate of ACT with a higher degree of precision, this study provides initial information and reassurance with regard to the effectiveness of ACT used at the community level under real life conditions of use. In this sense, findings of this study provide further evidence to support scaling-up implementation of HMM with ACTs.

## Competing interests

The authors declare that they have no competing interests.

## Authors' contributions

All the authors except SC and KM conceived the study; IA, ENB, and FB were principal investigators for their respective country's study site and together with CH, CF, GOG, BY, and SB participated in the research design and supervised data collection from the field. CH and DY were involved with the PCR analysis. BY, KM and SC contributed to data analysis and interpretation. FP, the WHO/TDR HMM research program manager monitored the three sites. All authors contributed to the draft. All authors read and approved the final manuscript.

## References

[B1] World Health Organization Guidelines for the treatment of malaria. WHO/HTM/MAL/20061108.

[B2] Falade C, Makanga M, Premji Z, Ortmann CE, Stockmeyer M, de Palacios PI (2005). Efficacy and safety of artemether-lumefantrine (Coartem^®^) tablets (six-dose regimen) in African infants and children with acute, uncomplicated falciparum malaria. Trans R Soc Trop Med Hyg.

[B3] Mulenga M, VangGeertruyden JP, Mwananyanda L, Chalwe V, Moerman F, Chilengi R, Van Overmeir C, Dujardin JC, D'Alessandro U (2006). Safety and efficacy of lumefantrine-artemether (Coartem^®^) for the treatment of uncomplicated *Plasmodium falciparum *malaria in Zambian adults. Malar J.

[B4] Dorsey G, Staedke S, Clark TD, Njama-Meya D, Nzarubara B, Maiteki-Sebuguzi C, Dokomajilar C, Kamya MR, Rosenthal PJ (2007). Combination therapy for uncomplicated falciparum malaria in Ugandan children: a randomized trial. JAMA.

[B5] Mutabingwa TK, Anthony D, Heller A, Hallett, Ahmed J, Drakeley C, Greenwood BM, Whitty CJM (2005). Amodiaquine alone, amodiaquine – sulfadoxine – pyrimethamine, amodiaquine – artesunate, and artemether-lumefantrine for outpatient treatment of malaria in Tanzanian children: a four-arm randomised effectiveness trial. Lancet.

[B6] Piola P, Fogg C, Bajunirwe F, Biraro S, Grandesso F, Ruzagira E, Babigumira J, Kigozi I, Kiguli J, Kyomuhendo J, Ferradini L, Taylor W, Checchi F, Guthmann JP (2005). Supervised versus unsupervised intake of six-dose artemether-lumefantrine for treatment of acute, uncomplicated *Plasmodium falciparum *malaria in Mbarara, Uganda: a randomised trial. Lancet.

[B7] World Health Organization Facts on ACTS (Artemisinin-based combination therapies), Geneva January 2006 update. http://www.rbm.who.int/cmc_upload/0/000/015/364/RBMInfosheet_9.htm.

[B8] World Health Organization (2005). The Roll Back Malaria strategy for improving access to treatment of malaria through home management of malaria. WHO/HTM/MAL/2005/1101.

[B9] Pagnoni F, Convelbo N, Tiendrebeogo J, Cousens S, Esposito F (1997). A community based programme to provide prompt and adequate treatment of presumptive malaria in children. Trans R Soc Trop Med Hyg.

[B10] Kidane G, Morrow RH (2000). Teaching mothers to provide home trea*tment of malaria in Ti*gray, Ethiopia: a randomized trial. Lancet.

[B11] Salako LA, Brieger WR, Afolabi BM, Umeh RE, Agomo PU, Asa S, Adeneye AK, Nwankwo BO, Akinlade CO (2001). Trea*tment of childhood fe *vers and other illnesses in three rural Nigerian communities. J Trop Paed.

[B12] Agyepong IA, Ansah E, Gyapong M, Adjei S, Barnish G, Evans D (2002). Strategies to improve adherence to recommended chloroquine treatment regimes: a qu*asi-experim *ent in the context of integrated primary health care delivery in Ghana. Soc Sci Med.

[B13] Sirima SB, Konaté A, Tiono AB, Convelbo, Cousens S, Pagnoni F (2003). Early treatment of childhood fevers with pre-packed antimalarial drugs in the home reduces severe malaria morbidity in Burkina Faso. Trop Med Int Health.

[B14] Ajayi IO, Falade CO, Bamgboye EA, Oduola AMJ, Kale OO (2008). Assessment of a treatment guideline to improve home management of malaria in children in rural south west Nigeria. Malar J.

[B15] D'Alessandro U, Talisuna A, Boelaert M (2005). Should artemisinin-based combination treatment be used in the home-based management of malaria?. Trop Med Int Health.

[B16]  Pagnoni F, Kengeya-Kayondo J, Ridley R, Were R, Nafo-Traoré F, Namboze J, Sirima S (2005). Artemisinin-based combination treatment in home-based management of malaria (Letter). Trop Med Int Health.

[B17] Hopkins H, Talisuna A, Whitty CJM, Sarah G, Staedke SG (2007). Impact of home-based management of malaria on health outcomes in Africa: a systematic review of the evidence. Malar J.

[B18] Ajayi IO, Browne EN, Garshong B, Bateganya F, Yusuf B, Agyei-Baffour P, Doamekpor L, Balyeku A, Munguti K, Cousens S, Pagnoni F (2008). Feasibility and acceptability of artemisinin-based combination therapy for the home management of malaria in four African sites. Malar J.

[B19] Appawu M, Owusu-Agyel S, Dadzie S, Asoala V, Anto F, Koram K, Rogers W, Nkrumah F, Hoffman SL, Fryauff DJ (2004). Malaria transmission dynamics at a site in northern Ghana proposed for testing malaria vaccines.

[B20] Awolola TS, Ibrahim K, Okorie T, Koekemoer LL, Hunt RH, Coetzee M (2003). Species composition and biting activities of anthropophilic *Anopheles mosquitoes *and their role in malaria transmission in a holo-endemic area of southwestern Nigeria. African Entomology.

[B21] Okello PE, Bortel WV, Byaruhanga AM, Correwyn A, Roelants P, Talisuna A, D'alessandro U, Coosemans M (2006). Variation in malaria transmission intensity in seven sites throughout Uganda. Am J Trop Med Hyg.

[B22] Plowe CV, Djimde A, Bouare M, Doumbo O, Wellems TE (1995). Pyrimethamine and proguanil resistance-conferring mutations in Plasmodium falciparum dihydrofolate reductase: polymerase chain reaction methods for surveillance in Africa. Am J Trop Med Hyg.

[B23] Mugittu K, Adjuik M, Snounou G, Ntoumi F, Taylor W, Mshinda H, Olliaro P, Beck HP (2006). Molecular genotyping to distinguish between recrudescents and new infections in treatment trials of *Plasmodium falciparum *malaria conducted in Sub-Saharan Africa: adjustment of parasitological outcomes and assessment of genotyping effectiveness. Trop Med Int Health.

[B24] MMV/WHO (2008). Methods and techniques for clinical trials on antimalarial drug efficacy. Informal consultation organized by the Medicines for Malaria Venture and cosponsored by the World Health Organization 29–31 May 2007, Amsterdam, The Netherlands Report published by MMV/WHO.

[B25] Hopkins H, Bebell L, Kambale W, Dokomajilar C, Rosenthal PJ, Dorsey G (2008). Rapid diagnostic tests for malaria at sites of varying transmission intensity in Uganda. J Infect Dis.

[B26] Rolland E, Checchi F, Pinoges L, Balkan S, Guthmann JP, Guerin PJ (2006). Operational response to malaria epidemics: are rapid diagnostic tests cost-effective?. Trop Med Int Health.

[B27] Kachur SP, Schulden J, Goodman CA, Kassala H, Elling BF, Khatib RA, Causer LM, Mkikima S, Abdulla S, Bloland PB (2006). Prevalence of malaria parasitemia among clients seeking treatment for fever or malaria at drug stores in rural Tanzania, 2004. Trop Med Int Health.

[B28] World Health Organization Antimalarial drug combination therapy. Report of WHO technical consultation, 4–5 April 2001 (WHO/CDS/RBM/200135).

[B29] Kabanywanyi AM, Mwita A, Sumari D, Mandike R, Mugittu K, Abdulla S (2007). Efficacy and safety of artemisinin-based antimalarial in the treatment of uncomplicated malaria in children in Southern Tanzania. Malar J.

[B30] Depoortere E, Guthmann J, Presse J, Sipilanyambe N, Nkandu E, Balkan S, Eldin de Pecoulas P, Legros D (2005). Efficacy and effectiveness of the combination of sulfadoxine/pyrimethamine and a 3-day course of artesunate for the treatment of uncomplicated falciparum malaria in a refugee settlement in Zambia. Trop Med Int Health.

[B31] Checchi F, Piola P, Fogg P, Bajunirwe F, Biraro S, Grandesso F, Ruzagira E, Babigumira J, Kigozi I, James Kiguli J, Kyomuhendo1 K, Laurent Ferradini L, Taylor WRJ, Guthmann J (2006). Supervised versus unsupervised antimalarial treatment with six-dose artemether-lumefantrine: pharmacokinetics and dosage-related findings from a clinical trial in Uganda. Malar J.

[B32] Ezzet F, vanVugt M, Nosten F, Looareesuwan S, White NJ (2000). Pharmacokinetcis and pharmacodynamics of lumefanthrine (benflumetol) in acute falciparum malaria. Antimicrobial Agents Chemother.

[B33] Cattamanchi A, Kyabayinze D, Hubbard A, Rosenthal PJ, Dorsey G (2003). Distinguishing Recrudescence from re-infection in a longitudinal antimalarial drug efficacy study: Comparison of results based on genotyping of MSP-1, MSP-2, and GLURP. Am J Trop Med Hyg.

[B34] Happi CT, Gbotosho GO, Sowunmi A, Falade CO, Akinboye DO, Gerena L, Kyle DE, Milhous W, Wirth DF, Oduola AMJ (2004). Molecular analysis of *Plasmodium falciparum *recrudescent malaria infections in children treated with chloroquine in Nigeria. Am J Trop Med Hyg.

[B35] Adjuik M, Babiker A, Garner P, Olliaro P, Taylor W, White N, International Artemisinin Study Group (2004). Artesunate combinations for treatment of malaria: meta-analysis. Lancet.

[B36] Nsungwa-Sabiiti J, Tomson G, Pariyo G, Ogwal-Okeng J, Peterson S (2005). Community effectiveness of malaria treatment in Uganda – a long way to Abuja targets. Ann Trop Paed.

[B37] Krause G, Sauerborn R (2000). Comprehensive community effectiveness of health care. A study of malaria treatment in children and adults in rural Burkina Faso. Ann Trop Paediatr.

[B38] Mueller O, Razum O, Traore C, Kouyate B (2004). Community effectiveness of chloroquine and traditional remedies in the treatment of young children with falciparum malaria in rural Burkina Faso. Malar J.

